# Establishment and application of a TaqMan-based quantitative PCR assay for simultaneous detection of bovine *Brucella* spp. and *Mycobacterium* spp.

**DOI:** 10.3389/fmicb.2025.1633809

**Published:** 2025-09-12

**Authors:** Shuai Zhang, Hui Zhao, Qiuju Guo, Ruixue Xue, Zixin Jiang, Wenduo Jiang, Linlin Xing, Xinhui Wei, Youxiang Diao, Yi Tang, Zouran Lan, Yue Zhang

**Affiliations:** ^1^College of Animal Medicine, Shandong Agricultural University, Tai'an, Shandong, China; ^2^Shandong Provincial Key Laboratory of Zoonotic Diseases, Jinan, Shandong, China; ^3^Jinan Nursing Vocational College, Jinan, China; ^4^Shandong Provincial Center for Animal Disease Provention and Control (Shandong Provincial Center for Zoonoses Epidemiology Investigation and Surveillance), Jinan, Shandong, China; ^5^Beijing Animal Husbandry and Veterinary Research Institute, Chinese Academy of Agricultural Sciences, Beijing, China

**Keywords:** *Brucella*, *Mycobacterium*, real-time PCR, TaqMan probe, simultaneous detection

## Abstract

Brucellosis and tuberculosis are two zoonotic, chronic infectious diseases caused by bacteria of the genus *Brucella* and *Mycobacterium*, respectively, which pose significant hazards to both animal husbandry and human health. Currently, mixed infections of these two pathogens are prevalent in livestock production; thus, establishing a molecular diagnostic method for the simultaneous detection and analysis of brucellosis and tuberculosis is crucial for the prevention and control of these diseases. By utilizing conserved regions within the genomes of *Brucella* and *Mycobacterium*, we designed specific primers and probes. After optimizing the developed qPCR assay conditions, we determined the lower limit of detection to be ten copies/ μL. Cross-testing with other bovine-derived pathogens demonstrated no cross-reactivity. Repeatability tests indicated that the coefficient of variation for the developed qPCR assay was less than 4.10% both within and between batches. We employed both the developed qPCR assay and a commercial qPCR assay to analyze sixty mixed infection samples of *Brucella* and *Mycobacterium* from various regions. The results revealed positivity rates of 100% and 96.67% for *Brucella*, and 100% and 95.00% for *Mycobacterium*, respectively. These findings indicate that a highly sensitive, specific, reproducible, and versatile qPCR method has been developed for the simultaneous quantitative detection of *Brucella* and *Mycobacterium*, which can be applied in studying the pathogenesis and epidemiology of these pathogens.

## 1 Introduction

Brucellosis, caused by *Brucella* infection, is a zoonotic systemic infectious disease primarily responsible for abortion, retention of the fetal coat, stillbirths, and weak calves in ewes, as well as testicular and epididymitis and arthritis in bulls (Dawood et al., 2023; Yang et al., 2023). The bacterium was first identified and isolated by the Scottish pathologist and microbiologist (David, 1887). The disease often manifests as a chronic or latent infection that can be transmitted via aerosols and poses a potential biological threat (Wang et al., 2021). Tuberculosis is a chronic infectious disease affecting both humans and animals, caused by bacteria of the genus *Mycobacterium*, characterized by tuberculous nodular granulomas in tissues and organs, alongside necrotic foci of caseation and calcification (Ramos et al., 2020; Reis et al., 2021). This disease can occur year-round, with a higher incidence observed in housed cattle. Factors such as overcrowding, darkness, dampness, poor hygiene, excessive labor and milking, and inadequate nutrition can facilitate the occurrence and spread of the disease (Santos et al., 2020; Pozo et al., 2022). Dairy cows and buffaloes are particularly susceptible. Transmission primarily occurs through the respiratory and digestive tracts, but can also occur via the placenta or during mating (Santos et al., 2022). Tuberculosis-infected animals serve as the main source of infection, with the tuberculosis bacillus distributed throughout various organ foci (Jalili et al., 2020; Perez-Morote et al., 2020). Diseased animals can excrete pathogens through feces, milk, urine, and tracheal secretions, contaminating the surrounding environment and facilitating the spread of infection (Dorn-In et al., 2020; Barnes et al., 2023). Brucellosis and tuberculosis are critically important to public health concerning livestock products, as they not only jeopardize livestock production but also pose serious risks to human health (Martinez-Guijosa et al., 2020; Zai et al., 2021). Therefore, the detection of both pathogens is essential in the study of bovine brucellosis and Mycobacteria (Allen et al., 2021; Zhang et al., 2025).

Rapid detection methods are crucial for the effective control of brucellosis and *Mycobacterium* infections (Pereira et al., 2022; Qin et al., 2024). In recent years, a quantitative real-time PCR (qPCR) has been developed that facilitates the accurate and reproducible quantification of gene copies (Ginzinger, 2002). This method has been extensively utilized to quantify the genomic copies of pathogenic microorganisms. qPCR has emerged as a powerful alternative in microbiological diagnostics due to its simplicity, rapidity, reproducibility, and high sensitivity when compared to other diagnostic methods (Chen et al., 2020).

Various detection methods for *Brucella* and *Mycobacterium* have been documented in the literature, including PCR techniques targeting the *Brucella Bcsp31* gene and the *Mycobacterium pncA* and *RD1* genes (Abdel-Hamid et al., 2021). Mascarenhas et al. validated qPCR technology for detecting *Mycobacterium bovis* and *Brucella abortus* in raw cow's milk (Mascarenhas et al., 2020). While these methods can identify pathogenic factors associated with *Brucella* and *Mycobacterium*, they exhibit significant limitations due to the complex and chaotic infection dynamics of these pathogens (Pinto et al., 2024; Kurmanov et al., 2022). Specifically, existing detection methods are unable to simultaneously detect the pathogenic factors of both *Brucella* and *Mycobacterium*; they can only assess each pathogen individually (Gabbassov et al., 2021; Rerkyusuke et al., 2024). Consequently, designing a method for the simultaneous detection of pathogenic factors from both *Brucella* and *Mycobacterium* is of paramount importance for advancing research in this area.

This study aims to establish test methods for the rapid detection of brucellosis and tuberculosis in dairy cows, assess the contamination status of fresh milk, and trace transmission routes. To achieve this, we designed specific primers and probes targeting the conserved regions of the *Brucella Omp25* and *Mycobacterium Mpt83* genes, and developed a dual qPCR method utilizing TaqMan probes. This method will be widely used in the detection of *Brucella* and *Mycobacterium*, laying the foundation for disease diagnosis and epidemiological investigations.

## 2 Materials and methods

### 2.1 Strains and clinical samples

The strains of *Brucella* spp., *Mycobacterium* spp., *Escherichia coli, Salmonella, Streptococcus, Staphylococcus*, and *Pasteurella multocida* utilized in this study were preserved in the laboratories of the Centre for Prevention and Control of Animal Diseases in Shandong Province. Milk, blood, and vaginal swab samples from animals suspected of brucellosis and tuberculosis were collected at cattle farms in a specific region of China. Positive clinical samples were isolated, identified, and stored by the China Animal Health and Epidemiology Centre.

### 2.2 Specific primers and probes

In this study, specific primers and probes were designed based on the conserved regions of the *Brucella Omp25* gene and the *Mycobacterium Mpt83* gene. The DNASTAR MegAlign software was used to align the Omp25 gene and the Mpt83 gene, respectively, to identify conserved regions and verify the specificity of the designed probes and primers.

### 2.3 DNA extraction and qPCR assay

Total DNA was extracted using the Bacterial Genomic DNA Extraction Kit (50 preps, Catalog DP302; TIANGEN, Beijing, China) following the manufacturer's instructions. All DNA templates were stored at −80°C until use. The developed qPCR assay was performed in a 25 μL reaction system (Gold Star Probe Mixture; CWBIO, CW0932M, Beijing, China), which included 12.5 μL of 2 × Gold Star Probe Mixture, 0.5 μL of *Brucella* forward primer (10 μM), 0.5 μL of *Brucella* reverse primer (10 μM), 0.5 μL of *Mycobacterium* forward primer (10 μM), 0.5 μL of *Mycobacterium* reverse primer (10 μM), 0.5 μL of *Brucella* probe (10 μM), 0.5 μL of *Mycobacterium* probe (10 μM), 7.5 μL of ddH_2_O, 1.0 μL of *Brucella* DNA template, and 1.0 μL of *Mycobacterium* DNA template. The developed qPCR assay was conducted using the Gentier 96 E/96 R Fully automated medical PCR analysis system. The reaction conditions included an initial denaturation at 95°C for 10 m, followed by forty cycles of denaturation at 95°C for 15 s and annealing/extension at 60°C for 1 m. During the extension step, fluorescent signals were collected.

### 2.4 Optimization of conditions for the developed qPCR assay

In this study, we optimized the concentrations of primers and TaqMan-probes, as well as the annealing temperature, to achieve the lowest Ct value with high fluorescence intensity. Six concentrations (0.1 M, 0.2 M, 0.4 M, 0.6 M, 0.8 M, and 1.0 M) suitable for primers and probes were tested, with three replicates for each concentration, while keeping other factors constant. After identifying the optimal concentrations of primers and TaqMan-probes, we evaluated five different annealing temperatures (56°C, 58°C, 60°C, 62°C, and 64 °C), again with three replicates for each temperature group. The optimal annealing temperature was selected based on the assay results.

### 2.5 Standard plasmid preparation, construct standard curves, and sensitivity

The *Omp25* gene (85 bp) of *Brucella* was amplified using specific forward (*Brucella*-F) and reverse (*Brucella*-R) primers. Similarly, the *Mpt83* gene (85 bp) of *Mycobacterium* was amplified using designated forward (*Mycobacterium*-F) and reverse (*Mycobacterium*-R) primers. The resulting PCR products were cloned into the plasmid vector pUC57 and subsequently verified through sequencing. The validated plasmids, pUC57-*Omp25* and pUC57-*Mpt83*, were purified using the Rapid Plasmid Miniaturization Kit (TIANGEN, DP105, Beijing, China) and quantified with a De Novix DS-11 spectrophotometer. A 10-fold dilution of the plasmids, pUC57-*Omp25* and pUC57-*Mpt83*, was prepared in 10 × Tris-EDTA buffer (pH 7.4) for the construction of a standard curve and to ascertain the detection limit of the developed qPCR assay.

### 2.6 Specificity analysis of the developed qPCR assay

In this study, the specificity of the developed qPCR assay was validated in triplicate using the following bovine bacterial species: *B. abortus, M. bovis, E. coli, Salmonella, Streptococcus, Staphylococcus*, and *P. multocida*.

### 2.7 Repeatability analysis of the developed qPCR assay

The dilutions of pUC57-*Omp25* and pUC57-*Mpt83*, with concentrations ranging from 1.0 × 10^8^ – 1.0 × 10^2^ copies/μL, were assessed over three consecutive days, with each day's assay repeated three times. The reproducibility of the developed qPCR assay was evaluated by calculating the intra- and inter-batch Coefficients of Variation (CV) based on the assay results.

### 2.8 Clinical samples detection

Sixty clinical samples suspected of *Brucella* and *Mycobacterium* infections were analyzed using the developed qPCR assay in this study and commercial kits. These samples were sourced from various regions, including Shandong, Inner Mongolia, and Henan. Sterile Phosphate-Buffered Saline (PBS) served as a control in the experiments.

### 2.9 Statistical analysis

Statistically significant differences in mean detection rates were determined using one-way ANOVA, conducted with Graph Pad Prism version 6. A *p-*value < 0.05 was considered significant, while a *p-*value < 0.01 was regarded as extremely significant.

## 3 Results

### 3.1 Primers and probes analysis

**Figure 1:**
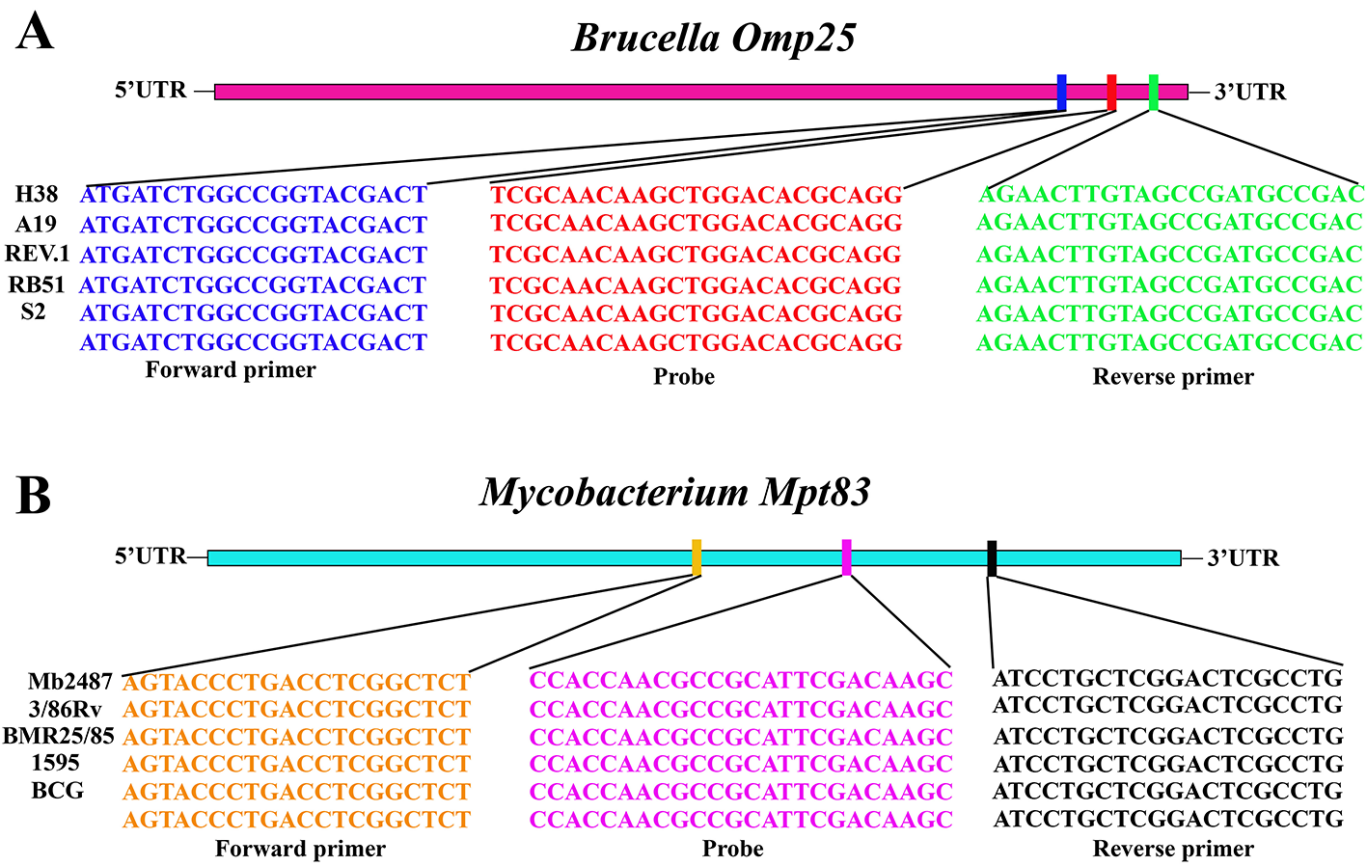
Result of sequence analysis. The results show that the primers and probes are situated within highly conserved regions of the *Brucella Omp25* gene and the *Mycobacterium Mpt83* gene. **(A)** The *Brucella Omp25* gene. **(B)** The *Mycobacterium Mpt83* gene.

The probes and primers utilized in this study were specifically designed based on the *Omp25* gene of *Brucella* and the *Mpt83* gene of *Mycobacterium*, as detailed in Table 1. Sequence analysis confirmed that the primers and probes are situated within highly conserved regions of the *Brucella Omp25* gene and the *Mycobacterium Mpt83* gene, as illustrated in Figure 1. Notably, the primers and probes exhibit a perfect match with *Brucella* and *Mycobacterium* sequences, demonstrating 100% homology to these sequences.

**Table 1:** Primers and probes used in this study.

**The developed qPCR assay**	**Oligo**	**Sequence (5^′^-3^′^)**	**Length (bp)**	**Positions (segment)**
*Brucella*	Forward primer	ATGATCTGGCCGGTACGACT	20	566–585 (Omp25)
	Probe	FAM-TCGCAACAAGCTGGACACGCAGG-TAMRA	23	588–610 (Omp25)
	Reverse primer	AGAACTTGTAGCCGATGCCGAC	22	642-640 (Omp25)
*Mycobacterium*	Forward primer	AGTACCCTGACCTCGGCTCT	20	289–308 (Mpt83)
	Probe	CY5-CCACCAACGCCGCATTCGACAAGC-BHQ3	24	380–403 (Mpt83)
	Reverse primer	ATCCTGCTCGGACTCGCCTG	20	484–503 (Mpt83)

### 3.2 The developed qPCR assay

**Figure 2:**
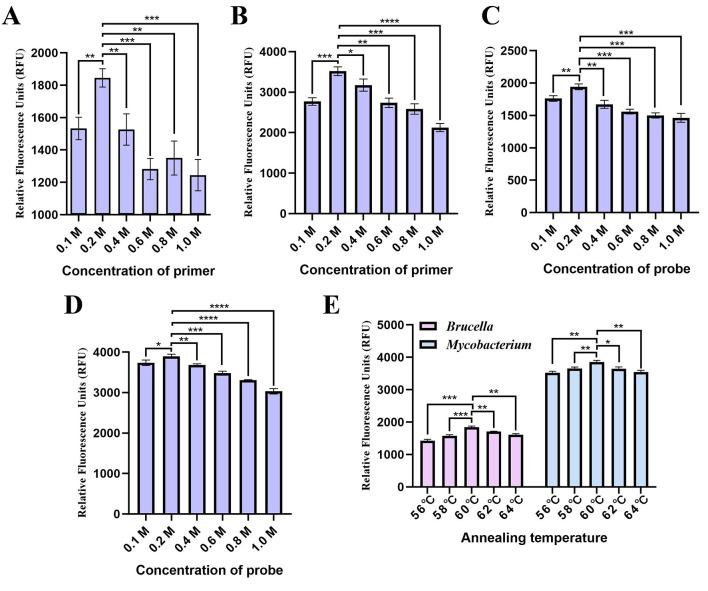
Optimization of the developed qPCR assay conditions. **(A)** Primer and **(B)** probe concentrations for the *Brucella* qPCR. **(C)** Primer and **(D)** probe concentrations for the *Mycobacterium* qPCR. **(E)** Annealing temperature.

The concentrations of primers and probes that resulted in high fluorescence were established (Figures 2A-D). The optimized volume for the developed qPCR assay reaction was set at 25 μL, comprising 12.5 μL of 2 × Gold Star Probe Mixture, 0.5 μL of the *Omp25* forward primer, 0.5 μL of the *Omp25* reverse primer, 0.5 μL of the *Omp25* probe, 0.5 μL of the *Mpt83* forward primer, 0.5 μL of the *Mpt83* reverse primer, 0.5 μL of the *Mpt83* probe, 1.0 μL of the *Omp25* DNA template, 1.0 μL of the *Mpt83* DNA template, and 7.5 μL of ddH_2_O. Although acceptable amplification was observed at temperatures ranging from 56°C to 64°C, the optimal conditions for the developed qPCR assay were determined to be at 60°C (Figure 2E).

### 3.3 Establishment of the standard curves

**Figure 3:**
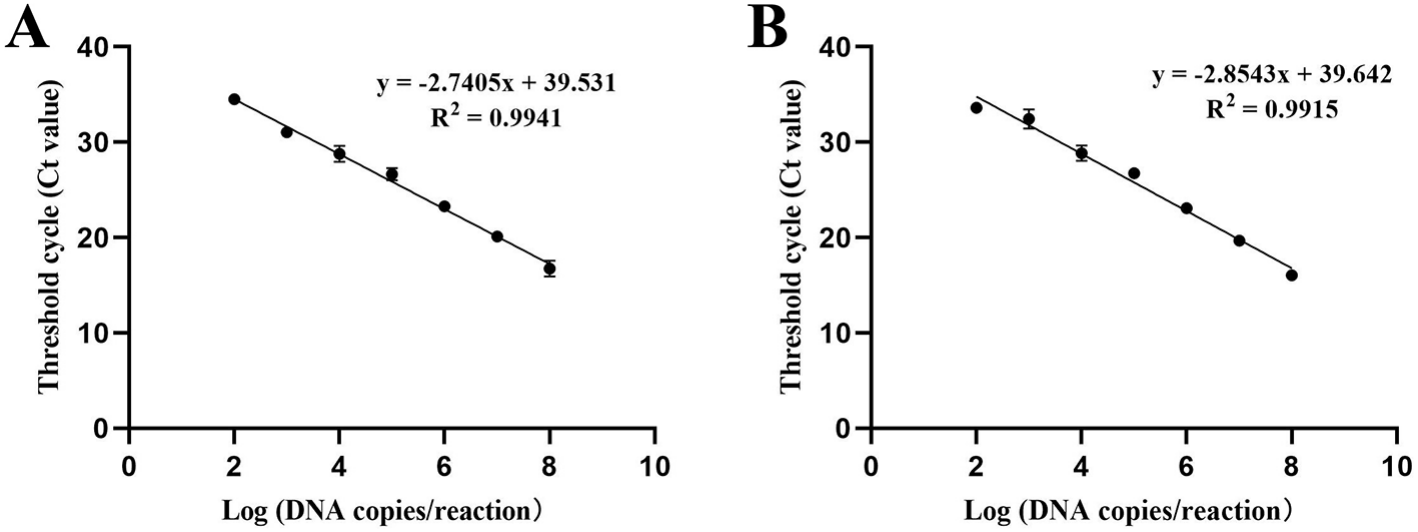
Standard curve of the the developed qPCR assay. The triplicate standard curve plots indicate a linear correlation between the Log of the copy number and the Cycle Threshold Value (CT). The logarithm values of the detected concentrations of the DNA standards (X axis) ranged from 1.0 × 10^8^ to 1.0 × 10^2^ copies/μ L, and used the corresponding Threshold cycle (CT value) of each reaction tube fluorescent signal approaching the set threshold (Y axis) of the amplification to perform linear regression. Three replicates were tested for each dilution. **(A)** Standard curve of *Brucella*. **(B)** Standard curve of *Mycobacterium*.

The triplicate standard curve plots demonstrate a linear correlation between the logarithm of the copy number and the cycle threshold (CT) values, as illustrated in Figure 3. The *Brucella* standard curve equation is given by Y = – 2.7405X + 39.531, where Y represents the threshold cycle and X denotes the logarithm of the standard. The linear correlation coefficient (*R*^2^) for this standard curve is 0.9941 (Figure 3A). Similarly, the *Mycobacterium* standard curve is represented by the equation Y = – 2.8543X + 39.642, with Y as the threshold cycle and X as the logarithm of the standard. The linear correlation coefficient *(R*^2^*)* for the *Mycobacterium* standard curve is 0.9915 (Figure 3B).

### 3.4 Sensitivity of the developed qPCR assay reaction

**Figure 4:**
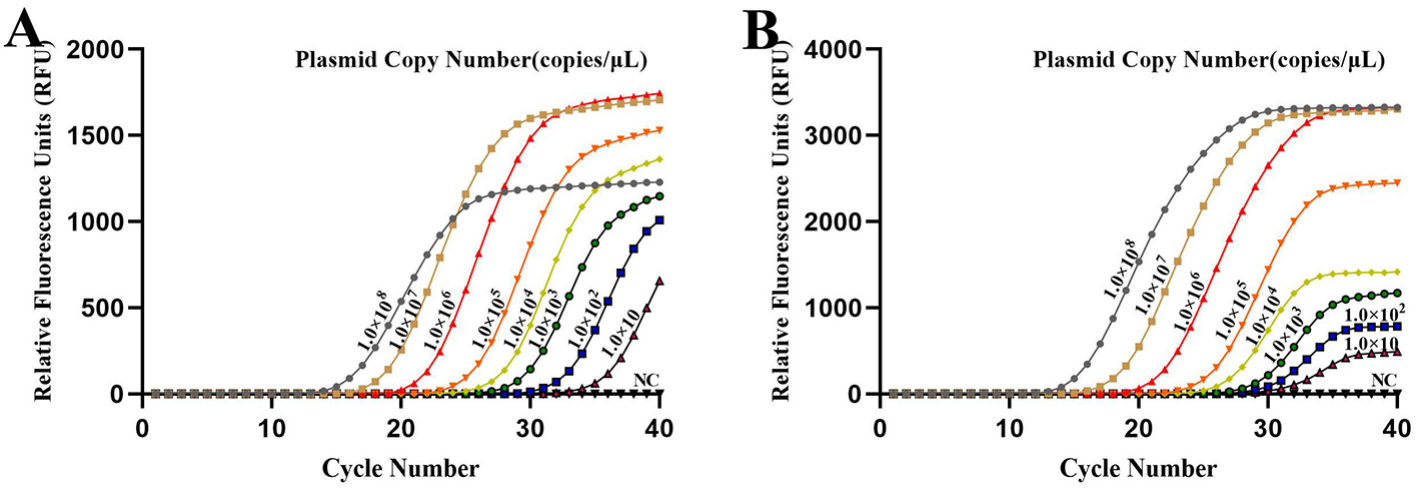
Sensitivity of the developed qPCR assay targeting **(A)** *Brucella* and **(B)** *Mycobacterium*. Sensitivity was assessed using serial dilutions of DNA standard plasmids, with a limit of detection of 1.0 × 10 copies/μL. NC, Nuclease-free water.

The sensitivity of the developed qPCR assay was evaluated by diluting the DNA standard plasmid from 1.0 × 10^9^ copies/ μL to 1.0 × 10^0^ copies/ μL. The results indicated that the assay's lowest limit of detection was 1.0 × 10 copies/ μL (Figure 4).

### 3.5 Specificity of the developed qPCR assay reaction

**Figure 5:**
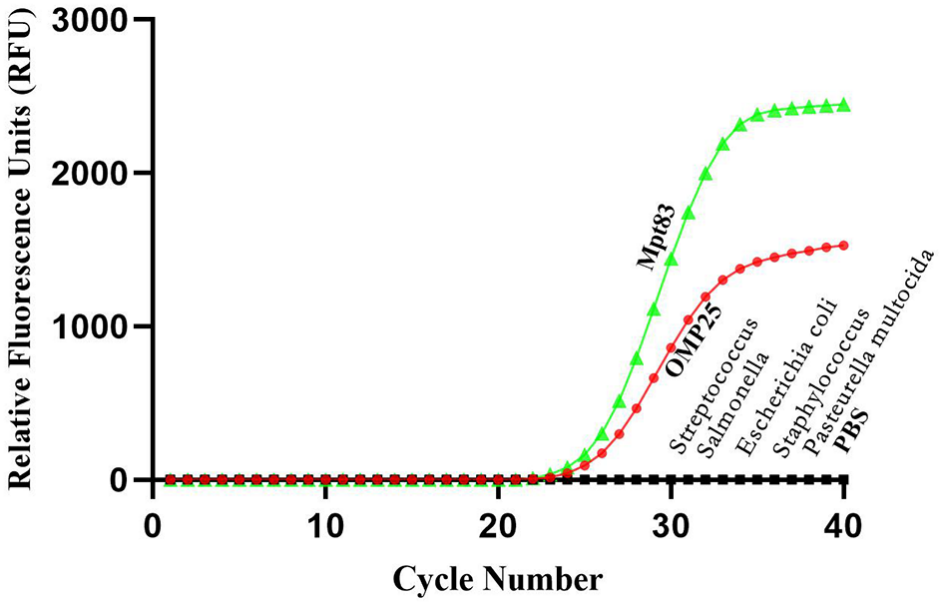
Specificity test results of real-time PCR assay using different strains. The results showed that this method can effectively distinguish *Brucella* and *Mycobacterium* from other pathogens.

In this study, five distinct bovine pathogens were utilized to evaluate the specificity of the developed qPCR assay detection. PBS served as the negative control. Strong fluorescent signals were observed in reactions involving *Brucella* and *Mycobacterium*, while the signals from the other three pathogen samples and the PBS control remained at baseline levels under the optimized reaction conditions. Consequently, *Brucella* and *Mycobacterium* were effectively distinguished from the other pathogens based on the variation in signal intensity (Figure 5).

### 3.6 Repeatability of the developed qPCR assay

The intra- and inter-assay reproducibility was assessed in triplicate over three different days using 10-fold serial dilutions of standard plasmid DNA, ranging from 1.0 × 10^8^-1.0 × 10^2^ copies. The results indicated that the intra-assay and inter-assay Coefficients of Variation (CV) for the *Brucella* qPCR method were 0.72 to 4.10 and 0.70 to 2.96, respectively (Table 2). Similarly, the intra-assay and inter-assay CVs for the *Mycobacterium* qPCR method were 1.17 to 2.49 and 0.89 to 2.78, respectively (Table 3). These findings suggest that the developed qPCR assay exhibits high reproducibility.

**Table 2:** Intra- and inter-assay variability of Ct values of assay in detection of *Brucella*.

**Copies of standard plasmid DNA**	**Intra-assay variability of Ct values**				**Inter-assay variability of Ct values**			
	**Proportion of positive samples** ^a^	**Ct**			**Proportion of positive samples** ^a^	**Ct**		
		**Mean**	**SD**	**CV (%)**		**Mean**	**SD**	**CV (%)**
10^8^	1.00	16.75	0.69	4.10	1.00	17.27	0.39	2.26
10^7^	1.00	20.11	0.30	1.49	1.00	20.59	0.29	1.41
10^6^	1.00	23.29	0.25	1.07	1.00	24.01	0.71	2.96
10^5^	1.00	26.63	0.53	1.98	1.00	26.97	0.19	0.70
10^4^	1.00	28.78	0.67	2.33	1.00	29.04	0.61	2.10
10^3^	1.00	31.06	0.22	0.72	1.00	32.14	0.54	1.68
10^2^	1.00	34.50	0.41	1.19	1.00	35.12	0.55	1.57

^a^Proportion of positive= positive samples/total tested samples (*n* = 3).

**Table 3:** Intra- and inter-assay variability of Ct values of assay in detection of *Mycobacterium*.

**Copies of standard plasmid DNA**	**Intra-assay variability of Ct values**				**Inter-assay variability of Ct values**			
	**Proportion of positive samples** ^a^	**Ct**			**Proportion of positive samples** ^a^	**Ct**		
		**Mean**	**SD**	**CV (%)**		**Mean**	**SD**	**CV (%)**
10^8^	1.00	16.06	0.19	1.17	1.00	16.91	0.47	2.78
10^7^	1.00	19.69	0.37	1.88	1.00	20.05	0.19	0.95
10^6^	1.00	23.08	0.38	1.67	1.00	23.47	0.21	0.89
10^5^	1.00	26.76	0.38	1.41	1.00	27.59	0.73	2.65
10^4^	1.00	28.86	0.66	2.29	1.00	29.18	0.64	2.19
10^3^	1.00	32.45	0.81	2.49	1.00	33.02	0.83	2.51
10^2^	1.00	33.63	0.41	1.23	1.00	34.16	0.79	2.31

^*a*^Proportion of positive= positive samples/total tested samples (*n* = 3).

### 3.7 Clinical sample application

Sixty clinical samples (milk, blood, vaginal swabs) from animals co infected with *Brucella* and *Mycobacterium* from various regions of China, were evaluated using both the developed qPCR assay and commercial qPCR kits. The prevalence rates of brucellosis and *Mycobacterium* were found to be 96.67% and 95%, respectively, according to the commercial qPCR kits. In contrast, the prevalence rates determined by the developed qPCR assay were 100% for both brucellosis and *Mycobacterium* (Tables 4, 5). These findings suggest that the developed qPCR assay exhibits superior detection accuracy.

**Table 4:** Clinical samples test results for *brucellosis*.

**Result by**		**No. of samples (total, 60)**
**The developed qPCR assay**	**qPCR kit** ^a^	
Pos.^b^	Pos.	58
Neg.^c^	Neg.	0
Pos.	Neg.	2
Neg.	Pos.	0

^*a*^Commercialized qPCR kits for brucellosis, ^*b*^Pos, Positive, ^*c*^Neg, Negative.

**Table 5:** Clinical samples test results for *Mycobacterium*.

**Result by**		**No. of samples (total, 60)**
**The developed qPCR assay**	**qPCR kit** ^a^	
Pos.^b^	Pos.	57
Neg.^c^	Neg.	0
Pos.	Neg.	3
Neg.	Pos.	0

^*a*^ Commercialized qPCR kits for brucellosis, ^*b*^Pos, Positive, ^*c*^Neg, Negative.

## 4 Discussion

Brucellosis is a zoonotic infection caused by the gram-negative, partially intracellular bacterium *Brucella* (Okafor et al., 2022). This pathogen infects a wide range of hosts, including cattle, sheep, pigs, and other mammals (Khairullah et al., 2024). The primary manifestations of brucellosis in livestock include undulant fever, infertility, abortion, arthritis, and orchitis (Selim et al., 2019). In contrast, tuberculosis is a chronic zoonosis caused by *Mycobacterium*, characterized by progressive wasting, the formation of tuberculous nodules, and caseous necrotic foci in various tissues and organs (Lu et al., 2022; Rossi et al., 2020). Epidemiological investigations have revealed a cross-infection between *Brucella* and *Mycobacterium*, resulting in decreased milk production, lower annual calving rates, and reduced meat production (Loiseau et al., 2019; Bonilla-Aldana et al., 2023; Pellegrini et al., 2022). These factors lead to significant economic losses in the cattle industry and severely impact the development of the animal husbandry sector.

Currently, there is no efficient method for the simultaneous detection of *Brucella* and *Mycobacterium*; therefore, there is a significant need for a rapid, highly sensitive, and specific method for their simultaneous detection in both the bovine industry and the research community (Triguero-Ocana et al., 2020; Khoshnood et al., 2022; Zeineldin et al., 2023; Zheng et al., 2024). In this study, we established a dual TaqMan-based real-time PCR assay targeting the *Omp25* gene of *Brucella* and the *Mpt83* gene of *Mycobacterium*. Verified through a series of experiments, the developed qPCR assay demonstrates high sensitivity, specificity, and reproducibility. The limit of detection for both *Brucella* and *Mycobacterium* was determined to be 1.0 × 10 copies/ μL. The developed qPCR assay yielded a strong fluorescent signal exclusively for *Brucella* and *Mycobacterium*, with intra-assay and inter-assay variability measured at less than 4.10% and 2.78%, respectively. Sixty clinical samples (milk, blood, vaginal swabs) from animals co infected with *Brucella* and *Mycobacterium* from various regions of China were tested using the developed qPCR assay and commercial qPCR assays. The positivity rate for the developed qPCR assay test for infection was 100%, while the positivity rates for the commercial qPCR tests were 96.67% for *Brucella* and 95.00% for *Mycobacterium*, indicating that the sensitivity of the former is superior. The high sensitivity demonstrated by the developed qPCR assay in this study is significant in low-load infections, where there is a risk of false negative results. However, the use of the developed qPCR assay requires certain experimental conditions, which limits their applicability for on-site detection of pathogens. In addition, this study did not set up an Internal Amplification Control (IAC), so it cannot be ensured that the master mix was in an optimal amplification state and that there were no inhibitors in the reaction, which is a limitation.

In conclusion, we have clearly established and validated the developed qPCR assay for the quantification of *Brucella* and *Mycobacterium* due to its remarkable sensitivity, reproducibility, rapidity, versatility, and high-throughput potential compared to other diagnostic methods. This detection method has low intra- and inter-assay variability and does not show cross-reactivity with other animal bacteria. Furthermore, its sensitivity surpassed that of commercial qPCR assays. These findings suggest that the assay can be utilized to simultaneously quantify *Brucella* and *Mycobacterium* DNA from various regions, thereby contributing to epidemiological investigations of animals infected with *Brucella* and *Mycobacterium*.

## 5 Conclusion

The developed qPCR assay in this study serves as a reliable tool for the rapid and simultaneous detection of *Brucella* and *Mycobacterium* in clinical samples. This method will be beneficial for epidemiological investigations and outbreak surveillance of both *Brucella* and *Mycobacterium*.

## Data Availability

The original contributions presented in the study are included in the article/supplementary material, further inquiries can be directed to the corresponding authors.
